# Powassan Virus in *Ixodes scapularis* Tick Populations, Illinois, USA, 2025

**DOI:** 10.3201/eid3209.260812

**Published:** 2026-09

**Authors:** Aurora Marguccio, Lance E. Jones, Brian Wilm, Arif Ciloglu, Valeria Trivellone, Millon Blackshear, Tomas Rodriguez, April Holmes, Samantha Kerr, Chang-Hyun Kim, Chris M. Stone

**Affiliations:** Prairie Research Institute, University of Illinois Urbana-Champaign, Champaign, Illinois, USA (A. Marguccio, L.E. Jones, B. Wilm, A. Ciloglu, V. Trivellone, M. Blackshear, T. Rodriguez, C.-H. Kim, C.M. Stone); Faculty of Veterinary Medicine, Erciyes University, Kayseri, Türkiye (A. Ciloglu); Illinois Department of Public Health, Springfield, Illinois, USA (A. Holmes, S. Kerr)

**Keywords:** Powassan virus, *Ixodes scapularis*, flavivirus, encephalitis, ticks, viruses, encephalitis viruses, tickborne diseases, vector-borne infections, United States

## Abstract

Powassan virus is a tickborne flavivirus that can cause severe encephalitis in humans. After the first known human case in Illinois, USA, we detected Powassan virus in adult *Ixodes scapularis* ticks collected from 2 sites in northern Illinois during 2025. Phylogenetic analysis confirmed the virus as Powassan virus lineage II.

Powassan virus (POWV; Flaviviridae: *Orthoflavivirus*) is a member of the tick-borne encephalitis serocomplex of flaviviruses and, although rare, poses a serious public health risk. Common symptoms include encephalitis and meningitis. Infection often results in considerable sequelae and, in ≈10% of cases, can be fatal (*1*). Reports have documented the distribution of POWV in the United States through case reports and studies of ticks and vertebrate hosts (*1*–*3*). US regions with cases of recorded POWV include those in the mid-Atlantic, Northeast, and Midwest, and the past decade has seen a consistent rise in human cases (*4*).

POWV consists of 2 clinically indistinguishable lineages: lineage I (POWV prototype) and lineage II (deer tick virus [DTV]) (*5*,*6*). Lineage I is maintained predominantly in an enzootic cycle involving *Ixodes cookei* ticks and woodchucks (*Marmota monax*), whereas lineage II is maintained in a cycle involving *Ixodes scapularis* ticks and white-footed mice (*Peromyscus leucopus*) (*1*). The first case of POWV in Illinois occurred in 2025, involving a resident from Tazewell County, although exposure may have occurred out of state (*7*). In response, we expanded our tick surveillance to assess the presence and distribution of POWV in *I. scapularis* tick populations in central and northwestern counties in Illinois and determine the phylogenetic lineages of detected strains.

We performed tick drags during October and November 2025 in 14 Illinois counties (Figure 1). We selected outdoor recreational areas containing woodland habitats as sampling locations. Within each county, 1 or 2 sites were visited 1–3 times, with at least five 150-m transects per visit. We used a 1-m^2^ white cloth drag attached to a wooden dowel for collection, according to previously established methods (*8*), and placed ticks in vials containing 85% ethanol. We sampled a total of 21 sites across 14 counties for *I. scapularis* ticks. We identified ticks according to species, life stage, and sex by light microscopy and then placed them in RNAlater solution (Thermo Fisher Scientific, https://www.thermofisher.com) and stored at −80°C until processing.

**Figure 1:**
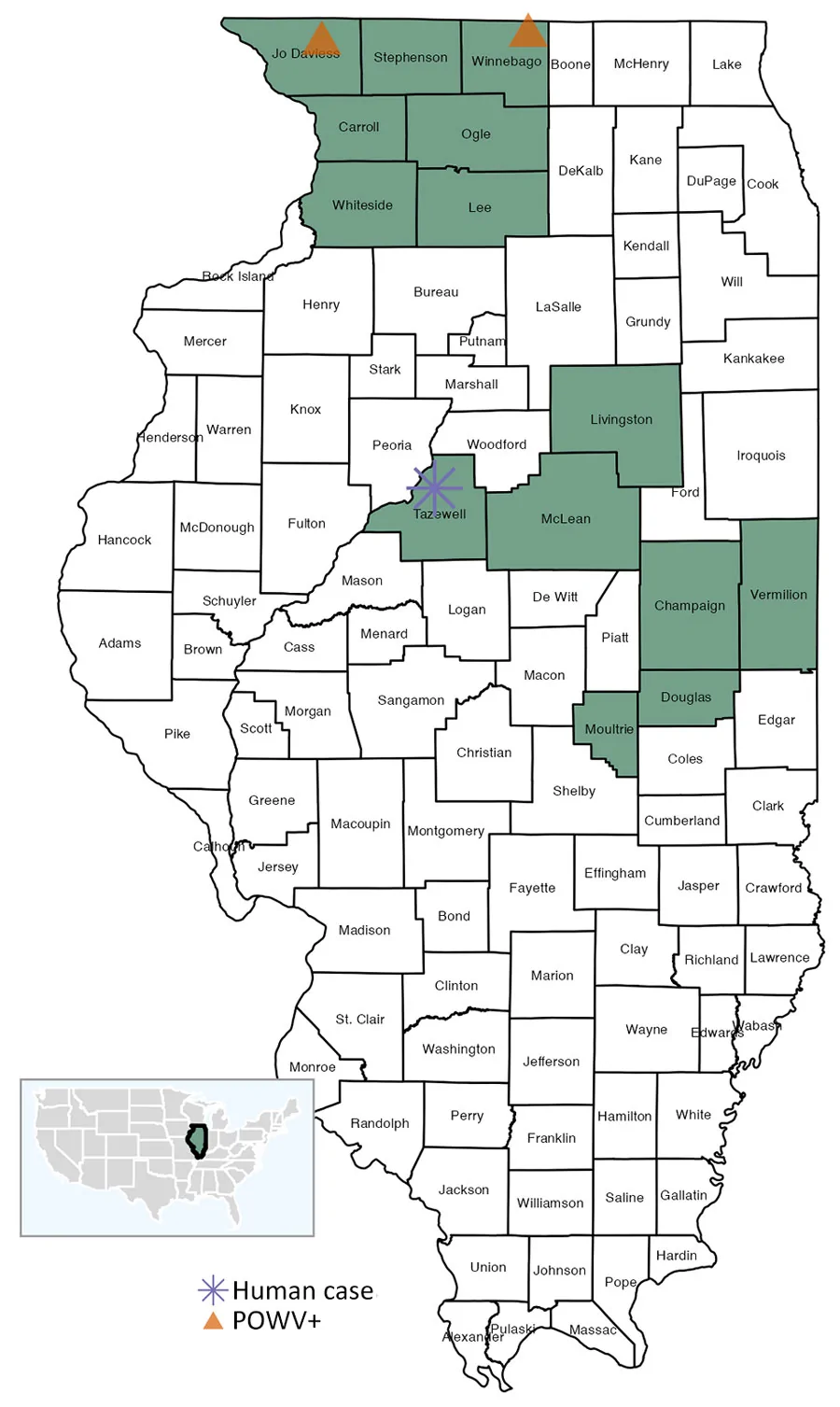
Geographic information from study of Powassan virus (POWV) in *Ixodes scapularis* tick populations, Illinois, USA, 2025. Green shading shows counties in Illinois where *I. scapularis* tick active surveillance included POWV testing. Orange triangles indicate locations where POWV-positive ticks were detected. Asterisk indicates county in which a human case was reported in 2025 (placed at random location within the county).

We extracted nucleic acids individually from ticks using the MagMAX Viral/Pathogen Ultra Nucleic Acid Isolation kit (Thermo Fisher Scientific) after crushing each specimen on liquid nitrogen. We screened extracts for POWV RNA presence by quantitative reverse transcription PCR (qRT-PCR) using an existing primer and probe set (*9*). We then reverse transcribed the extracts of qRT-PCR–positive samples (quantification cycle values <37) using the PrimeScript 1st strand cDNA Synthesis Kit (Takara Bio USA, https://www.takarabio.com) and amplified using an existing primer set (*5*). We slightly modified the reverse primer (5′-AGCGGGTGTTT TTCCGAGTCACWCA-3′) to detect both POWV lineage I and lineage II. When direct PCR failed, we ran a nested PCR using nested primer pairs POWVUTR-NestSeqF (5′-AGCATGACTGAACAGTCAAAAGA-3′) and POWVUTR-NestSeqR (5′-TTGTCAGGCTATCTGTGCC-3′). Amplicons were Sanger sequenced at the University of Illinois Urbana-Champaign Roy J. Carver Biotechnology Center (Champaign, Illinois, USA). We queried consensus sequences ranging 446–519 bp against the National Center for Biotechnology Information BLAST search tool (https://blast.ncbi.nlm.nih.gov). We considered only samples confirmed by both qRT-PCR and sequencing to be positive.

We generated sequence alignments using MAFFT (https://mafft.cbrc.jp/alignment/software) and a maximum-likelihood phylogenetic tree using IQ-TREE software (https://iqtree.github.io) with 1,000 bootstrap replicates for branch support. Phylogenetic reconstruction incorporated reference strains from various US states and localities in Canada and Russia (Appendix Table 1).

All collected ticks were adult *I. scapularis*, with the exception of a single *I. scapularis* nymph and a single *Dermacentor albipictus* male tick. We established density data for the female ticks collected (Appendix Table 2). We detected POWV in *I. scapularis* adults at single sites in Jo Daviess and Winnebago counties. The ticks determined to be positive for POWV (confirmed by sequencing) included 1 female and 2 male ticks from Winnebago collected on October 23, two male ticks from the same site collected on October 31, and 2 male ticks collected from a site in Jo Daviess on October 30. The prevalence of infection for adult *I. scapularis* ticks was 0.059 (Wilson CI 0.025–0.13) in Winnebago and 0.064 (Wilson CI 0.018–0.207) in Jo Daviess. POWV lineage I and lineage II reference strains were recovered as monophyletic clades (Figure 2), and the Illinois field samples clustered within lineage II. Within the DTV branch, we detected a cluster consisting of primarily northeastern United States samples, whereas Illinois samples clustered together with Wisconsin sequences.

**Figure 2:**
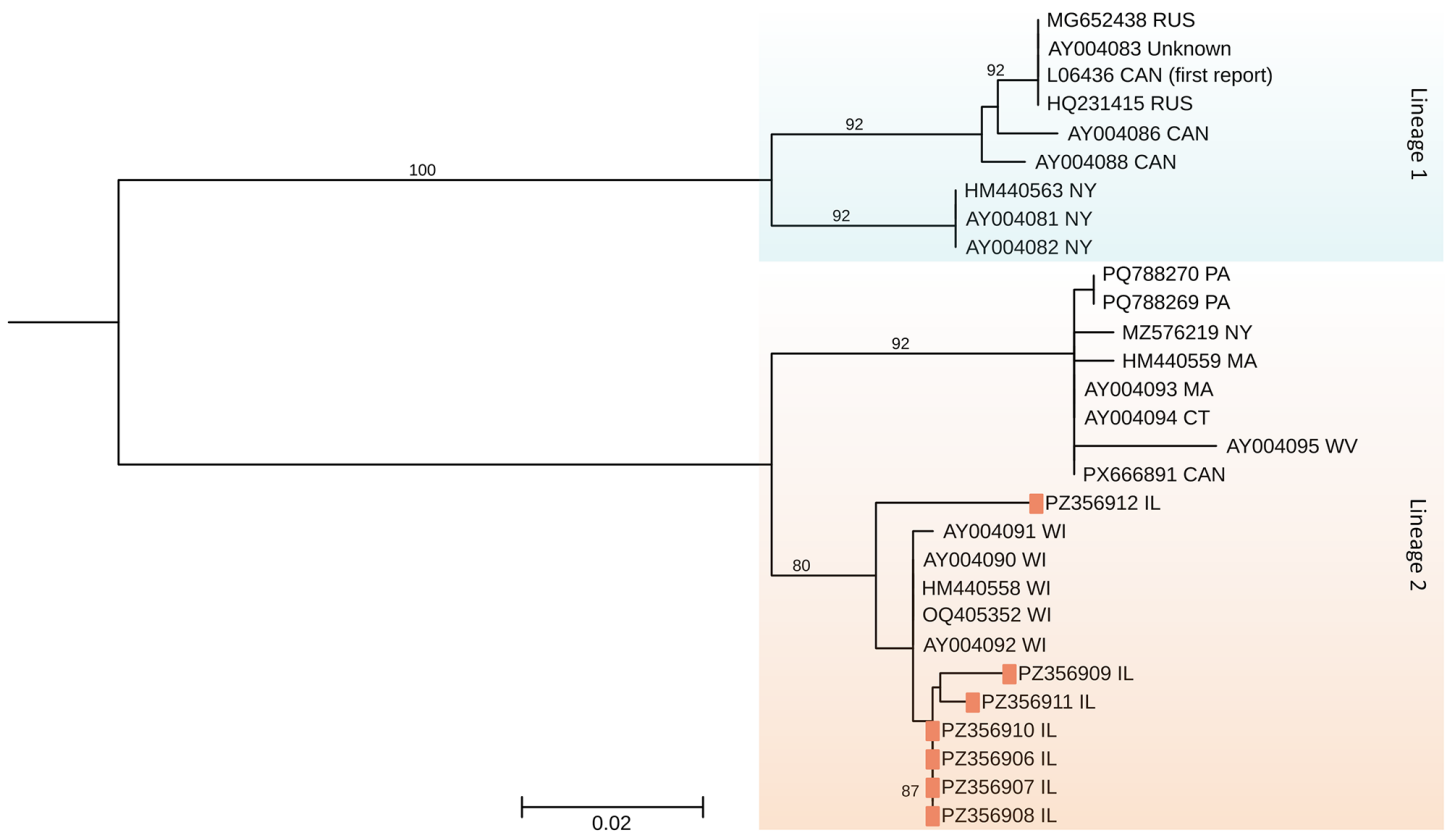
Maximum-likelihood phylogenetic tree of Powassan 3′ untranslated region created for study of Powassan virus in *Ixodes scapularis* tick populations, Illinois, USA, 2025. Branch lengths estimated using Hasegawa-Kishino-Yano substitution model with a gamma-distributed rate variation across 4 site categories. Bootstrap values >80% (1,000 replicates) are shown. Orange squares indicate samples from this study, clustering within lineage II together with samples previously detected in Wisconsin, USA. CAN, Canada; RUS, Russia.

Finding POWV-positive *I. scapularis* ticks in Illinois has relevance for healthcare providers, who may need to consider POWV exposure in establishing diagnoses, and also for health officials in crafting public health messaging to include such information as duration of tick attachment required for POWV transmission (*9*). The locations where we detected POWV-positive ticks were close to the Wisconsin border, where foci of POWV have previously been described (*1*). Of note, the prevalence in the positive counties was higher than that reported from a stable focus in Wisconsin, where the prevalence was 1.3% (*10*). An important question we pondered is whether POWV has been present, undetected, in Illinois for longer than the recent emergence our findings suggest. Increased surveillance and genomic analysis may elucidate POWV prevalence and phylogeographic history in this area of ongoing *I. scapularis* tick expansion.

Appendix Additional information for Powassan virus in *Ixodes scapularis* tick populations, Illinois, USA, 2025
