# Supplementary material for: Powassan Virus in Ixodes scapularis Tick Populations, Illinois, USA, 2025
Source: Emerg Infect Dis. 2026 Sep;32(9):1513–6. doi: 10.3201/eid3209.260812 (PMC13555739; doi:10.3201/eid3209.260812)
Supplement: Supplementary file 1 [file 26-0812-Techapp-s1.pdf]

Article DOI: http://doi.org/10.3201/eid3209.260812

EID cannot ensure accessibility for supplementary materials supplied by authors. Readers who have difficulty accessing supplementary content should contact the authors for assistance.

Powassan Virus in *Ixodes scapularis* Tick Populations, Illinois, USA, 2025

Appendix

Appendix Table 1. Powassan virus strains included in the phylogenetic analysis for a study of Powassan virus in *Ixodes scapularis* populations, Illinois, United States, 2025*

Strain designation	Geographic origin	Year of isolation	Source	Accession number
MG652438 RUS	Primorsky Krai, Russia	1975	Unknown	MG652438
AY004083 Unknown	Unknown	Unknown	Unknown	AY004083
L06436 CAN	Ontario, Canada	1958	Human brain	L06436
HQ231415 RUS	Russia	2006	Human brain	HQ231415
AY004086 CAN	Ontario, Canada	1964	*M. monax*	AY004086
AY004088 CAN	Ontario, Canada	1962	*T. hudsonicus*	AY004088
HM440563 NY	New York, USA	1964	Tick	HM440563
AY004081 NY	New York, USA	1964	Tick	AY004081
AY004082 NY	New York, USA	Unknown	Unknown	AY004082
PQ788270 PA	Philadelphia, USA	2020	*I. scapularis*	PQ788270
PQ788269 PA	Philadelphia, USA	2020	*I. scapularis*	PQ788269
MZ576219 NY	New York, USA	2020	*I. scapularis*	MZ576219
HM440559 MA	Massachusetts, USA	1996	*I. scapularis*	HM440559
AY004093 MA	Massachusetts, USA	1994	*I. dammini*	AY004093
AY004094 CT	Connecticut, USA	1994	*I. dammini*	AY004094
AY004095 WV	West Virginia, USA	1977	*Vulpes* spp.	AY004095
PX666891 CAN	Ontario, Canada	1997	Human brain	PX666891
AY004091 WI	Wisconsin, USA	1997	*I. dammini*	AY004091
AY004090 WI	Wisconsin, USA	1999	*I. dammini*	AY004090
HM440558 WI	Wisconsin, USA	1999	*I. scapularis*	HM440558
OQ405352 WI	Wisconsin, USA	NA	Plasmid clone	OQ405352
AY004092 WI	Wisconsin, USA	1999	*I. dammini*	AY004092
25–0057.4–1-F	Illinois, USA	2025	*I. scapularis*	PZ356912
25–0055.4–1	Illinois, USA	2025	*I. scapularis*	PZ356909
25–0056.6–4	Illinois, USA	2025	*I. scapularis*	PZ356911
25–0055.5–4-F	Illinois, USA	2025	*I. scapularis*	PZ356910
25–0024.1–1	Illinois, USA	2025	*I. scapularis*	PZ356906
25–0024.7–2	Illinois, USA	2025	*I. scapularis*	PZ356907
25–0024.8–2	Illinois, USA	2025	*I. scapularis*	PZ356908

*NA, not available

Appendix Table 2. Number of adult *Ixodes scapularis* collected over all site visits per county and density of females per 150 m^2 transect for a study of Powassan virus in *Ixodes scapularis* populations, Illinois, United States, 2025.

County	No. females collected	No. males collected	Density of females/150 m^2 (SE)
Carroll	11	9	0.45 (0.17)
Champaign	0	1	0 (0)
Douglas	1	2	0.07 (0.07)
Jo Daviess†	14	17	0.61 (0.23)
Lee	1	5	0.05 (0.05)
Livingston	0	0	0 (0)
McLean	6	7	0.24 (0.09)
Moultrie	0	0	0 (0)
Ogle	24	35	0.54 (0.14)
Stephenson	18	10	0.66 (0.23)
Tazewell	18	19	0.95 (NA)
Vermilion	3	1	0.14 (0.08)
Whiteside	5	7	0.24 (0.11)
Winnebago†	31	54	1.15 (0.36)

* NA, not available; SE, standard error.
†Powassan-positive tick locations.
